# Medullary colon cancer presenting with total necrosis of all regional lymph node metastases: morphologic description of a presumed immune-mediated event

**DOI:** 10.1186/s13000-014-0204-x

**Published:** 2014-10-22

**Authors:** Andrew Mitchell, Yves Bendavid

**Affiliations:** Department of Anatomic Pathology and Cytology, Maisonneuve-Rosemont Hospital, 5415 Boulevard de L’Assomption, Montreal, Quebec H1T 2M4 Canada; Department of Surgery, Maisonneuve-Rosemont Hospital, Montreal, Quebec Canada

**Keywords:** Medullary, Colon, Cancer, Necrosis, Lymph node, Metastases

## Abstract

Medullary carcinoma is a rare type of colon cancer with characteristic clinical and molecular features. Notably, despite its high-grade histology, the prognosis is generally better than for colonic adenocarcinoma of the usual type. We present herein a singular case of medullary colon cancer in which all of numerous lymph node metastases in the surgical resection specimen were completely necrotic in the face of a wholly viable primary tumor. Possible mechanisms are discussed with emphasis on immune-mediated factors.

**Virtual Slides:** The virtual slide(s) for this article can be found here: http://www.diagnosticpathology.diagnomx.eu/vs/13000_2014_204

## Background

Medullary cancer is a rare but well-characterized type of colon carcinoma [1,2]. Although displaying high-grade histologic features, in general there are fewer lymph node metastases and better overall survival compared to typical adenocarcinomas of the colon. We describe a singular case of previously untreated medullary colon cancer in which all lymph node metastases were entirely necrotic whereas the primary tumor was, in stark contrast, completely histologically viable. Although we cannot provide a definitive explanation for this phenomenon, an immune-mediated mechanism seems most likely.

## Case presentation

A 75 year old woman presented with diffuse pain in her arms and legs of recent onset. Neurologic consultation led to a diagnosis of polyneuropathy of uncertain etiology, likely paraneoplastic in origin. Her past medical history included hypothyroidism and idiopathic sensory neuronopathy (both of at least fifteen years duration), pernicious anemia, oculopharyngeal dystrophy, arterial hypertension, atherosclerotic heart disease, chronic obstructive pulmonary disease, and appendectomy. She had stopped smoking forty years previously.

As part of a subsequent “neoplastic workup” a PET-scan revealed a highly metabolically active mass in the cecum, with no other sites suspicious for neoplasia identified. A CT-scan of the thorax was negative. She was not anemic, and stated she was completely asymptomatic regarding the cecal lesion. An attempt at colonoscopic biopsy was unsuccessful, as, due to pain and significant diverticulosis, the colonoscope could not be passed beyond the sigmoid. Shortly thereafter, a laparoscopic right hemicolectomy was performed.

Postoperatively, the patient received standard chemotherapy for colorectal carcinoma. Nearly one year following surgery, there is no evidence of recurrent disease. Her polyneuropathy has resolved.

## Materials and methods

All formalin-fixed, paraffin-embedded tissue blocks from the surgical resection specimen were cut at 4 microns and routinely stained with hematoxylin-phloxin-saffranin (HPS). Selected tissue blocks were stained with the histochemical stain PAS with diastase for the detection of epithelial mucin and the following immunohistochemical markers: pancytokeratin (AE1/AE3, Millipore, 1:1000), cytokeratin Cam 5.2 (5D3, Becton Dickinson, 1:2), calretinin (SP65, Roche, prediluted), CEA (II-7, Dako, 1:1000), CDX-2 (EPR2764Y, Roche, prediluted), EBV (C1, Dako, 1:1000), MLH1 (M1, Ventana, prediluted), PMS2 (EPR 3949, Ventana, prediluted), MSH2 (G219-1129, Ventana, prediluted), and MSH6 (44, Ventana, prediluted). Mutation of the BRAF V600E gene was evaluated by polymerase chain reaction according to a standard protocol.

## Pathologic findings

Macroscopic examination of the right hemicolectomy specimen revealed a discoid cecal mass 4 cm in the largest dimension with invasion of the muscle wall. There was no infiltration of surrounding soft tissues or of the visceral peritoneum (serosa). Multiple firm, whitish, lymph nodes suggestive of metastatic tumor were readily found in the mesentery. The rest of the specimen was normal.

**Figure 1:**
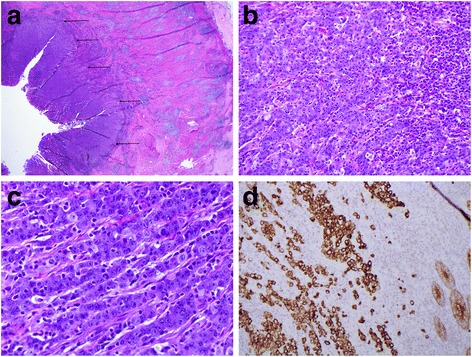
**Histology of the primary tumor. a)** The well-circumscribed primary medullary carcinoma is on the left (arrows). The caecal muscular wall (right) shows a marked Crohn’s-like inflammatory infiltrate. **b** and **c)** Tumor cells are arranged in cords with associated intra-tumoral lymphocytes. Gland formation is absent. There are high-grade cytologic features and several mitoses. Necrosis is absent. **d)** Pancytokeratin positivity of the tumor cells. Normal colonic epithelium at bottom right provides a positive internal control.

Microscopic examination demonstrated features typical of medullary cancer (MC) (Figure 1a, b, c). Tumor necrosis was absent. The tumor cells were positive with the epithelial markers pancytokeratine (Figure 1d) and Cam 5.2, calretinin, and CDX-2 (weak) in accordance with the diagnosis. CEA and EBV immunostaining were negative. PAS with diastase staining confirmed the absence of intra- or extracellular mucin. Microsatellite instability – high (MSI-high) was demonstrated by immunohistochemical staining: MLH1/PMS2 negative, MSH2/MSH6 positive. The BRAF V600E gene was mutated.

**Figure 2:**
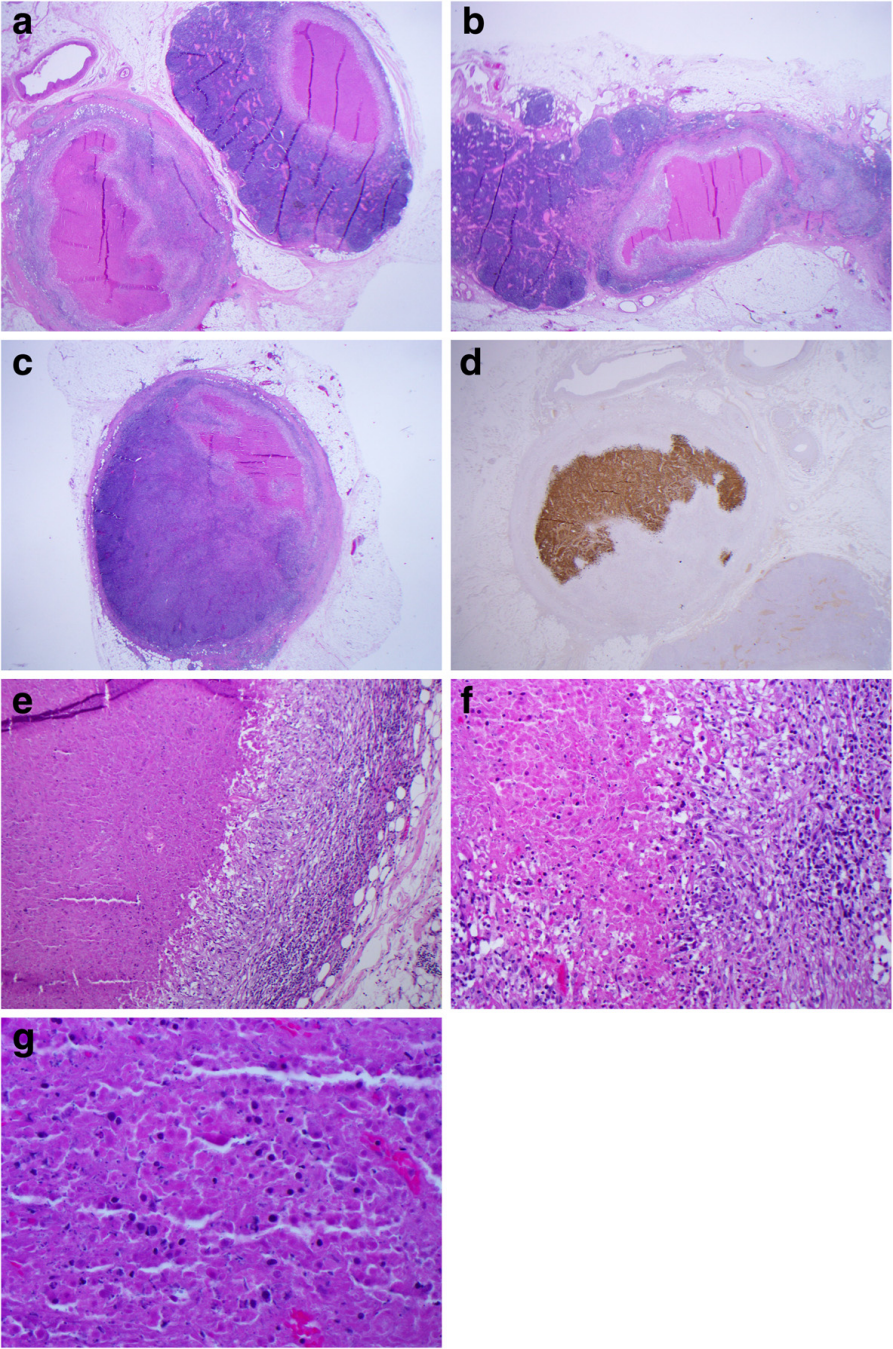
**Histology of the lymph node metastases. a, b and c)** Several lymph nodes showing necrotic metastases surrounded by granulomatous inflammation. Viable tumor cells are completely absent. **d)** Pancytokeratin positivity of the necrotic tumor cells within one of the lymph nodes. **e** and **f)** High power views of lymph nodes with necrotic tumor and associated granulomatous inflammation. Viable tumor cells are completely absent. **g)** High power view of necrotic tumor within lymph node. Viable tumor cells are completely absent.

Although no peritumoral lymphovascular invasion was seen, 11 of 32 resected peri-colic lymph nodes were positive. Staging of the tumor was therefore T2 N2b M0 [3]. However, all metastatic foci showed complete tumor necrosis surrounded by a brisk granulomatous inflammatory reaction. In none of the involved lymph nodes were any viable tumor cells found (Figure 2a, b, c, e, f, g). Staining with pancytokratine and Cam 5.2 showed strong positivity in multiple lymph nodes, confirming the epithelial nature of the necrotic foci (Figure 2d).

## Discussion

Medullary carcinoma (MC) of the colon is a rare tumor with characteristic histologic features representing 5-8/10,000 colon cancer cases. An analysis of all 50 cases of MC in the Surveillance, Epidemiology and End Results (SEER) database from 1973 to 2006 concluded that it occurs most commonly in the proximal colon (74%), favors older women, is less likely to feature lymph node metastases, and has a good prognosis with one and two year relative survival rates of 92.7% and 73.8% [1].

Macroscopically and microscopically, MC is well circumscribed with a “pushing” or “expanding” growth pattern. It often grows to a large size (the majority at diagnosis are greater than 7 cm) with infiltration of adjacent structures. The tumor cells have “high grade” cytologic features: high nuclear/cytoplasmic ratios, round to oval nuclei, large amphophilic nucleoli, and vesicular chromatin. Mitoses are common and apoptotic bodies are often found. The cells are arranged in nests, cords, and sheets and may widely infiltrate the intestinal wall; geographic necrosis and perineural and angiolymphatic invasion are common [2]. Intense intratumoral or peritumoral lymphocytic infiltrates, lymphocytic infiltrates at the advancing tumor margin and conspicuous “Crohn’s-like” lymphoid reactions are common [4]. Positivity with neuroendocrine immunohistochemical markers is found in approximately one third of cases [2].

Microsatellite instability – high (MSI-high): MLH1/PMS2 negative, MSH2/MSH6 positive is typical [5]. BRAF V600E mutation, as seen here, indicates a sporadic tumor [6].

The differential diagnosis of MC includes poorly differentiated colorectal adenocarcinoma, neuroendocrine carcinoma and “lymphoepithelioma-like carcinoma”, for which the differential diagnostic features are discussed elsewhere [2,7].

To the best of our knowledge the present case is unique, as concomitant with a wholly viable primary MC tumor, all numerous (11) lymph node metastases were completely necrotic at the time of surgery. In contrast, of the 68 MCs in the series of Wick et al [2] the authors stated “there was no difference in the microscopic appearance of lymph nodal tumor deposits vis-à-vis that of the primary neoplasms”. Also of interest, in another large study of MC only one of 50 cases (2%) had greater than 7 lymph node metastases [1].

Do these findings represent an example of “spontaneous tumor regression”? Criteria for the diagnosis of spontaneous regression were put forward nearly fifty years ago: 1) histologic regression of biopsy proven metastases, 2) radiologic regression of presumed neoplastic disease, and 3) regression of metastatic tumor after therapy considered ineffective [8]. The first criterion would most closely correspond to the histologic findings we describe. Given its incidence and prevalence, spontaneous regression of colorectal cancer is an extremely rare event, with only 21 cases reported between 1900 and 2005 according to a major review [8]. All examples were moderately to poorly differentiated adenocarcinomas of the usual type. Regression almost invariably involved the primary tumor or metastases following removal of the primary tumor. It should be noted, however, that in several cases where regression of metastatic disease was reported, regression, or not, of the primary tumor was not clearly specified.

Numerous hypotheses concerning the mechanism(s) of tumor regression have been proposed, none conclusive (8). Similarly, we cannot provide a precise explanation for this phenomenon, but the interplay of patient-specific factors and immune-mediated events is likely. Regarding patient factors, there had been no neoadjuvant therapy. Causes of local ischemic events such as bowel torsion (volvulus) or tissue entrapment in an internal hernia or by adhesions were not observed at surgery. However, the patient had several auto immune-mediated diseases (hypothyroidism, idiopathic sensory neuronopathy, and pernicious anemia), suggesting heightened activity of her immune system and, perhaps, increased immunosurveillance.

As the primary tumor was entirely viable, the potential role of the lymph node microenvironment in inducing tumor necrosis is worthy of consideration. One can speculate that tumor antigen processing by lymph node antigen presenting cells (APCs) may have instigated a localized immunologic response leading to widespread cell necrosis. This would imply, conversely, that the APCs infiltrating and surrounding the primary tumor itself were incapable of instigating such a response: the tumor cells metastatic to the lymph nodes were therefore likely viable.

Tumor cell necrosis, formally regarded as a passive phenomenon, is now considered a form of programmed cell death (type III PCD) [9]. Whereas apoptosis (type II PCD) involves the death of individual cells, necrosis involves large cell numbers. It is mediated by complex signaling pathways that are activated when, for example, inadequate vascularization leads to ischemia and hypoxia with resulting cell energy deprivation; a variety of anti-cancer drugs also induce necrosis. Tumor cell necrosis in turn further stimulates the immune system: the release of a variety of cytoplasmic molecules to the extracellular space upon loss of cell membrane integrity leads to activation of APCs and macrophages. Dendritic cell maturation and T-cell proliferation subsequently occur with optimization of tumor antigen presentation and phagocytosis of dead cells. [9]. As such, although the primary initiating event in our case is unknown, we propose that this “lymph node-limited tumor necrosis” may be due to the ability of lymph node specific immune cells to mount a tumor directed immune response.

Finally, regarding the patient’s polyneuropathy, the development of symptoms before tumor detection and the resolution of symptoms following tumor removal clinically support a paraneoplastic etiology [10]. However, no testing for neuro-oncologic antibodies was performed. Paraneoplastic neurological syndromes due to colon cancer are extremely rare, with sensory neuropathy and vasculitis having been described [11]. Of note, it has been observed that tumors causing paraneoplastic neurologic disorders are often “heavily infiltrated with inflammatory cells” and have a better prognosis than histologically identical tumors with no paraneoplastic neurologic manifestations [10].

## Conclusion

In summary, we present a unique case of medullary colon cancer. The simultaneous occurrence of necrotic lymph node metastases and a viable primary tumor is possibly explained by an immunologic response in the lymph node microenvironment. The patient’s history of multiple autoimmune diseases raises questions as to the role of her “activated” immune system in responding to the metastases. This case, albeit of morphologic interest, and perhaps representing a form of spontaneous regression, raises important questions relating to the immunologic response to tumor cells instigated within lymph nodes.

## Consent

Written informed consent was obtained from the patient for publication of this Case Report and any accompanying images. A copy of the written consent is available for review by the Editor-in-Chief of this journal.

## Abbreviations

APC: Antigen presenting cell
CT: Computerized tomography
MC: Medullary cancer
MSI: Microsatellite instability
PCD: Programmed cell death
PET: Positron emission tomography
